# Highly efficient visible light active ZnO/Cu-DPA composite photocatalysts for the treatment of wastewater contaminated with organic dye

**DOI:** 10.1038/s41598-023-43842-z

**Published:** 2023-09-30

**Authors:** Biniyam Abdu Berehe, Ayalew H. Assen, A. Santhana Krishna Kumar, Hidayath Ulla, Alemayehu Dubale Duma, Jia-Yaw Chang, Gangaraju Gedda, Wubshet Mekonnen Girma

**Affiliations:** 1 Department of Chemistry, College of Natural Science, Wollo University, P.O. Box 1145, Dessie, Ethiopia; 2 Department of Chemistry, National Sun Yat-sen University, No. 70, Lien-Hai Road, Gushan District, Kaohsiung, 80424 Taiwan; 3 Faculty of Geology, Geophysics and Environmental Protection, AGH University of Science and Technology, Al. Mickiewicza 30, 30-059 Krakow, Poland; 4 Department of Physics, School of Engineering, Presidency University, Bangalore, 560064 India; 5 Bio and Emerging Technology Institute (BETin), Nanotechnology Directorate, P.O. Box 5954, Addis Ababa, Ethiopia; 6 Department of Chemical Engineering, National Taiwan University of Science and Technology, Taipei, Taiwan, Republic of China; 7 Central Research Laboratory, K S Hegde Medical Academy, NITTE (Deemed to Be University), Deralakatte, Mangaluru, Karnataka 575018 India; 8 Department of Animal Science and Technology and BET Research Institute, Chung-Ang University, Anseong, Gyeonggi-do 17546 Republic of Korea

**Keywords:** Environmental chemistry, Nanoscale materials, Catalysis, Environmental chemistry, Materials chemistry

## Abstract

Industrial effluents are a leading major threat for water contamination, subsequently which results in severe health associated risks. Hence, purifying wastewater before releasing into the water resources is essential to avoid contamination. In this study, ZnO/Cu-DPA nano-composites were prepared by altering the percentage of Cu-DPA (20%, 30%, 40%, and 50% which are denoted to be ZnO/20%Cu-DPA, ZnO/30%Cu-DPA, ZnO/40%Cu-DPA and ZnO/50%Cu-DPA) using a simple mechanical grinding process. Several spectroscopic studies were employed such as electron paramagnetic analysis (EPR), powdered X-ray diffractometer (PXRD), UV–Vis absorbance spectroscopy, Fourier transform infrared (FT-IR) spectroscopy and scanning electron microscope to characterize these nano-composites. The photo-catalytic activities of the prepared nano-composites were studied by degrading MB under visible light irradiation. ZnO, ZnO/20%Cu-DPA, ZnO/30%Cu-DPA, ZnO/40%Cu-DPA and ZnO/50%Cu-DPA degradation efficiencies were determined to be 71.8, 78.5, 77.1, and 66.1%, respectively. Among the composite catalysts, the ZnO/20%Cu-DPA coupled system are demonstrated the best efficiency (87%) for photo-degradation of MB within 80 min when exposed to visible light. The ZnO/Cu-DPA nano-composites had a greater MB photodegradation efficiency than pristine ZnO owing to p-n heterojunction in the linked system. Under visible light irradiation, the ZnO/20%Cu-DPA catalysed the conversion of dissolved O_2_ to hydroxyl radicals (OH·), triggering the reduction of MB. This suggests that ·OH is the primary specific active radical involved in the photo-catalytic decomposition of MB. Furthermore, EPR analysis indicates the existence of ·OH in the photo-catalytic system. The proposed nano-composites (ZnO/20%Cu-DPA) reusability was investigated across three cycles as the most efficient photo-catalyst. The results show that, the ZnO/Cu-DPA nano-catalyst is a potential candidate for the remediation of dirty water.

## Introduction

Potable water is widely recognized as an essential living requirement on the Earth. Due to human activity, pollutants, including antibiotics, organic dyes, and other inorganic/organic compounds, have been introduced into water systems^1^. These toxins are hazardous to local ecosystems, aquatic resources, wildlife, and people and resistant to physical, chemical and biodegradation processes. Among the various pollutants, synthetic dyes are a significant category of water pollutants because they usually contain a plethora of aromatic ring configurations^2^. Therefore, their degradability is hindered by their complicated chemical structure. Thus, it is generally agreed that these chemicals are hazardous and persistent in the natural world^3^.

Previously, these organic dyes were eliminated from water/wastewater samples utilizing various procedures, including photocatalysis, membrane processes, adsorption, biodegradation and coagulation-flocculation^4–6^. Nevertheless, photocatalysis based on heterogeneous semiconducting materials is considered a distinct, efficient, cost-effective, and environmentally benign technology for efficiently eradicating a widespread range of inorganic/organic/pollutants^7^. When a powerful semiconductor, often oxides and sulphides of transition metals, has its electron–hole pairs (e−/h+), hydroxyl (·OH), and superoxide (·O_2_^−^) radicals photogenerated by exposure to ultraviolet (UV) or visible light^8^. These reactive species are potent chemicals that can oxidize or reduce pollutants, causing their molecules to fragment into smaller and smaller pieces. With additional assaults, degradation intermediates may be mineralized to H_2_O, CO_2_, and other organic or inorganic products. Many studies have been conducted to determine the precise process in this regard^7,9–11^. On the other hand, semiconductors based on metal oxides are extensively employed for degrading organic compounds via photocatalysis contaminants, including organic dyes^12^. ZnO, an n-type semiconductor, is one of the most crucial semiconductor photocatalysts because of its great photosensitivity and stability, low toxicity, wide availability, significant binding energy, excellent thermal conductivity, notable electron mobility, and economical and lesser toxicity^13,14^. Due to its large bandwidth of 3.2 eV, ZnO is limited to absorbing light in the near UV range, which is a significant limitation. Unfortunately, only 4–5% of UV radiation is emitted by sunlight^15,16^. As a result, using photocatalytic processes to maximize solar energy is still challenging.

Narrow bandgap semiconductors have a light sensitivity that extends significantly further into the visible spectrum. However, due to the recombination of the photo-induced electron–hole pairs, these semiconductors have a hard time keeping their photoactivity for extended periods. Narrow bandgap materials are frequently added to ZnO to boost its visible-light-driven photocatalytic effectiveness. Radical lifetimes, on the other hand, are often far shorter than reaction rates. As a result, we reasoned that adding a functional component to a photocatalytic system that could extend the free radical lifetime would considerably improve the system's photocatalytic performance. Furthermore, self-assembled metal complexes have been claimed to be effective molecular containers for temporary intermediate encapsulation and stability^16,17^. As a result, including metal complexes can potentially boost photocatalytic activity. In particular, studying how metal complexes affect the stability of free radicals created by light irradiation could pave the way for creating a revolutionary technique.

The coordination bonds between transition metal cations and organic ligands define crystalline solids known as metal complexes. The metal complexes have distinct advantages in terms of performance, including tuneable pore size and pore surface, low density, and high surface area^18^. In recent studies, semiconductor materials such as ZnO^19^ have been combined with metal complexes to increase their photocatalytic efficiency.

Cu-based metal complexes are the most preferred of all the reported metal complexes because of the high availability and stability of copper on the Earth, which makes them excellent sacrificial templates for creating catalysts with large surface area and porosity. Additionally, copper may generate a variety of geometric complexes with a variety of ligands^20^. In 2022, Teerawat et al*.* developed a Cu(II)–Quinoline complex immobilized on a Silica support for the degradation of MB dye. The reported Cu(II) complex acted as a Photo-Fenton-like catalyst showing the efficiency of 95% for the dye degradation in 2.5 h^21^. Same year Hemant et al. reported copper(II) Schiff base metal complex as a powerful candidate for the photocatalytic degradation of MB dye in the presence of H_2_O_2_ as an oxidising agent^22^. In 2020, Samira et al. reported four mononuclear copper(II) coordinated complexes showing photocatalytic degradation of various dyes such as Rhodamine B (RhB), Congo red (CR), crystal violet (CV), methyl orange (MO), and MB dye^23^. All the complexes showed 100% of dye degradation using hydrogen peroxide as oxidant in 90 min. Same year Murugaiyan et al. studied 4-hydroxy benzohydrazide-grafted biopolymer Schiff base Cu(II) complexes as eco-friendly catalysts for the photocatalytic degradation of MB dye^24^. In 2019, Harshita et al. synthesized a multifunctional Cu(II) pyridyl complex (CP1) for the rapid elimination of MB and RhB through the adsorption as well as photocatalytic degradation process^25^. The catalyst (0.3 g L^−1^) was able to decolorize 95.8% of MB (16 mg L^−1^) and 93.7% of RhB (23.95 mg L^−1^) in 35 min.

Inspired from the above interesting concept, we propose a ZnO/Cu-DPA nano-composite showing significantly high photocatalytic activity in comparison to an individual of ZnO and Cu-DPA. In this study, four ZnO/Cu-DPA nanocomposites were prepared by altering the percentage of Cu-DPA (20%, 30%, 40%, and 50% which are denoted to be ZnO/20%Cu-DPA, ZnO/30%Cu-DPA, ZnO/40%Cu-DPA, and ZnO/50%Cu-DPA) using a simple mechanical grinding process. The photo-catalytic activities of the prepared catalysts were studied by degrading MB under visible light irradiation. ZnO, ZnO/20%Cu-DPA, ZnO/30%Cu-DPA, ZnO/40%Cu-DPA and ZnO/50%Cu-DPA degradation efficiencies were determined to be 71.8, 78.5, 77.1, and 66.1%, respectively. Among the composite catalysts, the ZnO/20%Cu-DPA coupled system demonstrated the best efficiency (87%) for the photo-degradation of MB dye within 80 min when exposed to visible light. To the best of our knowledge, no research has been conducted on wastewater MB dye degradation using the ZnO/Cu-DPA composite catalyst. Additionally, ZnO/Cu-DPA composite catalysts could improve photocatalytic activity by broadening the light absorbance region, reducing the recombination of electrons and holes, and increasing catalyst porosity due to Cu-DPA complexes.

## Materials and methods

### Chemicals and reagents

Potassium hydroxide (KOH, 99.8%), isopropanol (99%), Ethylenediaminetetraacetic acid (EDTA, 99.8%), Zinc sulphate hexahydrated (ZnSO_4_·6H_2_O, 98%), Copper sulphate pentahydrated (CuSO_4_·5H_2_O, 99%), Copper nitrate mono hydrate (Cu(NO_3_)_2_·H_2_O, 99%), diphenylamine (98%), methylene blue (C_16_H_18_ClN_13_S, 99%), ethanol (99.8%) and5,5-Dimethyl-1-Pyrroline-N-Oxide (DMPO, ≥ 98%). Analytical-grade chemicals were employed in this work. Reagents and solvents are utilized without purification.

### Synthesis of Cu-DPA complex

Cu-DPA complex was prepared by following the reported in the literature with a little modification^26^. 3 g of diphenylamine and 3 g of CuSO_4_·5H_2_O were dissolved in 15 mL of ethanol and distilled water, respectively. For 2 h, the two solutions were mixed and swirled on a hot plate using a magnetic stirrer to obtain a homogeneous solution. The resulting solution was allowed to sit for three days to allow the formation of a blue precipitate. Following centrifugation separation, the precipitates were washed four times using deionized water and ethanol and then dried in a drying oven at 40 °C for 24 h. The resulting precipitate was given the designation Cu-DPA and stored for further use.

### Synthesis of ZnO NPs

Direct precipitation was used to prepare ZnO NPs, as previously reported in the literature, with a few tweaks^27^. In a nutshell, a 0.2 M ZnSO_4_.6H_2_O aqueous solution and a 0.4 M KOH aqueous solution were each made up to 50 mL in volume. A white suspension was formed by adding the KOH solution slowly to the ZnSO_4_.6H_2_O solution while stirring vigorously at room temperature. After centrifuging at 5000 rpm for 20 min, the white product was washed three times with distilled water and once with 100% ethanol. We calcined the final product for 3 h at 500 °C.

### Synthesis of ZnO/Cu-DPA nanocomposites

ZnO/Cu-DPA nanocomposites were prepared by simple mechanical grinding method using different ratio of prepared ZnO NPs and Cu-DPA. The prepared ZnO NPs and Cu-DPA were mixed with a mass ratio of 80% /20%, 70% /30%, 60%/40% and 50%/50% by increasing the amount of Cu-DPA, respectively then grinding using pestle and mortar. The obtained result was calcined at 300 °C for 1 h.

### Characterization

Shimadzu X-ray diffractometer (XRD, Shimadzu XRD-7000) was employed for XRD investigation. The morphological structure was determined using Scanning electron microscopy with a field emitter (FESEM, JSM 6500F, JEOL), FT-IR spectrometer (PerkinElmer) was used to determine the functional groups. The MB dye degradation was carried out under a tungsten lamp reactor (Japan, 150W) and assessed by UV–Vis Spectrophotometer (Shimadzu-3600 Plus), and Bruker Elexsys E-580 spectrometer was used for the electron spin resonance (EPR) studies.

### Photocatalytic experiment

MB degradation was performed by following the reported literature^28^. To perform photodegradation investigations, the prepared suspensions were agitated for 30 min at dark to obtain the adsorption/desorption equilibrium stage. After that, 25 mg of the catalyst was added in a 250 mL beaker with 125 mL of MB aqueous solution (10 ppm, pH 7) and irradiated by a 150 W tungsten lamp 10 cm above the cell while being swirled on a magnetic stirrer and cooled with water.

A control sample without the catalyst was irradiated to study MB elimination by direct photolysis. At regular intervals, 5 mL of the suspension was collected and centrifuged at 6000 rpm, and the absorbance of the clear supernatant was measured using a UV–visible spectrophotometer at λ_max_ 663 nm. Absorbance was also evaluated on the purified solutions (A_o_ and A_t_ before and after irradiation, respectively). Furthermore, the reusability of the most efficient sample, ZnO/20%Cu-DPA photocatalyst, was examined by continuously observing the visible-light-induced photodegradation of MB. In particular, the catalyst (75 mg) was blended with MB (10 ppm) (375 mL) and agitated for 30 min. The resulting mixture was then subjected to 80 min of exposure to visible light. After that, an aliquot solution (5 mL) was taken out of the solution mixture. After each cycle, the photocatalyst was washed with deionized water and acetone to remove any residual solution. The sample was dried and then submerged in a second cycle of a fresh solution containing the same amount of MB as the first.

### Determination of active species

To further understand the photocatalytic mechanism, some additional experiments were also carried out with cupric nitrate (Cu(NO_3_)_2_), isopropanol and disodium ethylenediamine-tetraacetate (EDTA) as scavengers for electrons, ·OH and holes, respectively. 20 mL of isopropanol, EDTA, and Cu (NO_3_)_2_ at 10 mM concentrations were added to MB (10 ppm, 125 mL) containing 25 mg of ZnO/20%Cu-DPA catalyst. The mixture was then churned for 80 min in a visible light source reactor. As a final step, the absorbance was determined at the maximum wavelength of MB (λ_max_ = 663 nm).

## Results and discussion

**Figure 1:**
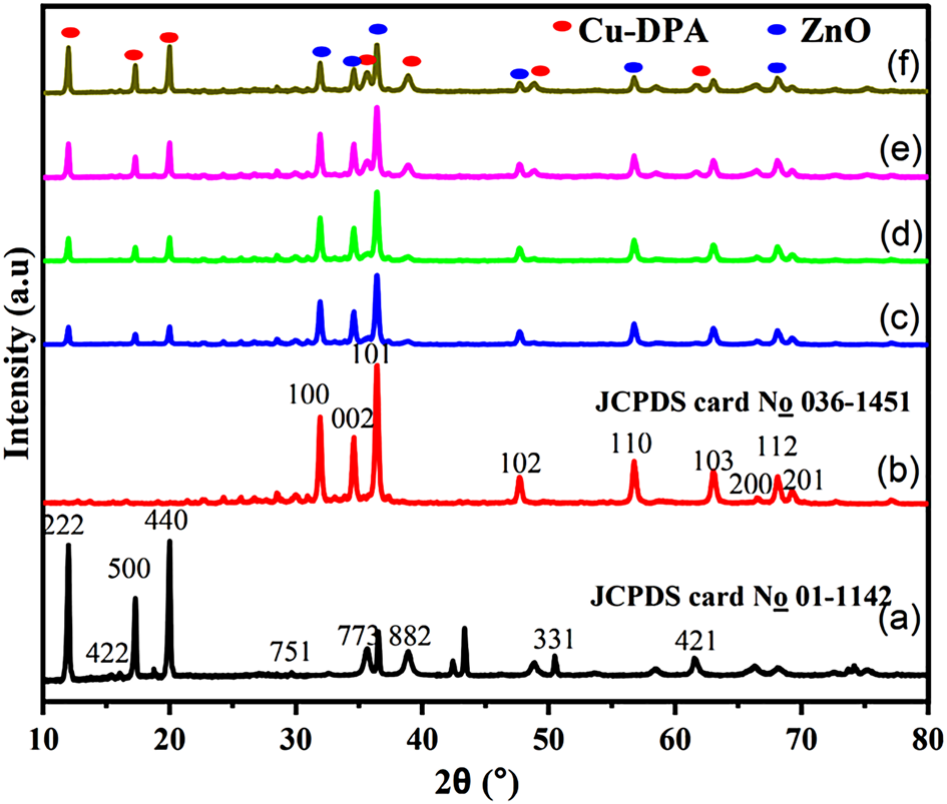
XRD patterns for (**a**) Cu-DPA, (**b**) ZnO, (**c**) ZnO/20% Cu-DPA, (**d**) ZnO/30% Cu-DPA, (**e**) ZnO/40% Cu-DPA and (**f**) ZnO/50% Cu-DPA.

X-ray diffraction investigations were performed at room temperature to investigate the crystal structure ZnO, Cu-DPA and ZnO/Cu-DPA composites (ZnO/20%Cu-DPA, ZnO/30%Cu-DPA, ZnO/40%Cu-DPA, and ZnO/50%Cu-DPA) (Fig. 1). The XRD pattern of the as-prepared Cu-DPA sample, shown in Fig. 1a, exhibited three prominent diffraction peaks with Bragg angles of 12.1°, 17.6°, and 20°. This strongly validates the crystalline character of the prepared complex and is consistent with the diffraction pattern of other Cu-based crystalline metal complexes^26,29,30^. In addition, XRD analysis of the Cu-DPA sample revealed peaks at 2θ values of 36.4, 42.2 and 61.3° (Figure S1) which are indicative of the formation of the by-product Cu_2_O^31^. It is vital to remember that XRD can detect crystalline compounds with crystallite sizes over 30 Å and crystallinity content over 3%^32^. The characteristic XRD peaks of Cu_2_O (Figure S1) infer that the Cu_2_O concentration is at least 3%^33^. The percentage of Cu_2_O calculated by the Rietveld refinement technique showed a Cu_2_O percentage of 6.9%. Typical diffraction peaks at 2θ angles of 31.8, 34.4, 36.3, 47.6, 56.6, 62.9, 66.4, 68.0, 69.2, and 77.0° confirmed the hexagonal wurtzite ZnO crystallite phase, which is consistent with previously reported ZnO NPs^34^. The XRD peaks were assigned to their corresponding reflection planes of (100), (002), (101), (102), (110), (103), (200), (112), (201), and (202), respectively. Moreover, as shown in Fig. 1b, the observed pattern for the synthesized ZnO NPs consisted of this conventional pattern with excellent conformity. Similarly, the XRD spectra (Fig. 1c–f) of ZnO/Cu-DPA composites revealed the presence of ZnO and Cu-DPA diffraction peaks. All the peaks for the constituent NPs are present in the pattern of the prepared composites, proving their viability.

Scherrer and Williamson-Hull (W–H) equations were also used to determine the crystallite size of uncoupled and coupled ZnO, Cu-DPA, and ZnO/Cu-DPA composites. The standard Scherrer equation is defined by Eq. (1), where the average crystallite size (*d*) is directly dependent on the X-ray wavelength (λ) and the Scherrer constant (k) and inversely dependent on the peak width of the diffraction peak (*β*). In this investigation, a Cu-K line (1.5406 Å) was used as the radiation source. The k-constant, which varies with crystallite shape, typically takes the value of 0.9, and θ equals half the diffraction angle^35,36^.

1 $$d = \frac{k\lambda }{{\beta \cos \theta }}$$

Crystallite sizes were estimated to be 40.67, 24.63, 24.14, 26.81, 29.80, and 29.87 nm for as-synthesized Cu-DPA, ZnO, ZnO/20% Cu-DPA, ZnO/30% Cu-DPA, ZnO/40% Cu-DPA, and ZnO/50% Cu-DPA, respectively. During the possible deformation observed in the samples, the *d*-spacing of a given *hkl* plane can shift. This shift in *d*-spacing may generate lattice strain and repositioning of the peak. Thus, inhomogeneous micro-strain caused by dislocation-like defects (strain broadening) and size broadening generated by coherent scattering volume shrinking can contribute to peak broadening in a diffraction peak^37^. The current investigation probed the additive effects of crystallite size and lattice strain on the broadening of diffraction peaks in as-synthesized samples using the following W–H equation^38^.

2 $$\beta \cos {\uptheta } = \frac{k\lambda }{d} + {\upeta }\sin {\uptheta }$$

The right-hand side of Eq. (2) displays the Scherrer equation, which shows how crystallite size affects peak broadening, while the second part, also known as Stokes and Wilson expression, verifies how internal strain in samples affects peak broadening. The width of a diffraction peak is commonly affected by factors like experimental parameters, crystal defects, strain changes of different grains, and crystallite size changes. The strain effect broadens when the value of (1/d) is increased, even though the broadening itself does not change. By the time the strain factor is close to zero, the W–H equation in Eq. (2) transforms into the net Scherrer equation. The W–H equation can be used to discriminate between size and strain effects, which is useful because they often co-occur during XRD peak broadening. A standard W–H plot will show a linear relationship between βcos(θ) and sin(θ), with an intercept of (kλ/*d*) and a slope of (η). The slope of a W–H plot might be either positive or negative. Positive slopes indicate tensile tensions, while negative slopes indicate compressive strains. The relative positions of peaks can also be altered by other factors, including contact and coherency stresses, triple junctions, grain boundaries, and stacking faults^39^. Figure S2 depicts the corresponding graph. Based on the intercept values of the W–H plots, the effective crystallite sizes of the Cu-DPA, ZnO, ZnO/20% Cu-DPA, ZnO/30% Cu-DPA, ZnO/40% Cu-DPA, and ZnO/50% Cu-DPA samples were determined to be 37.47, 37.47, 38.5, 46.21, 47.81, and 63.02 nm, respectively. Slopes in W–H plots indicate tensile strain, apart from Cu-DPA.

**Figure 2:**
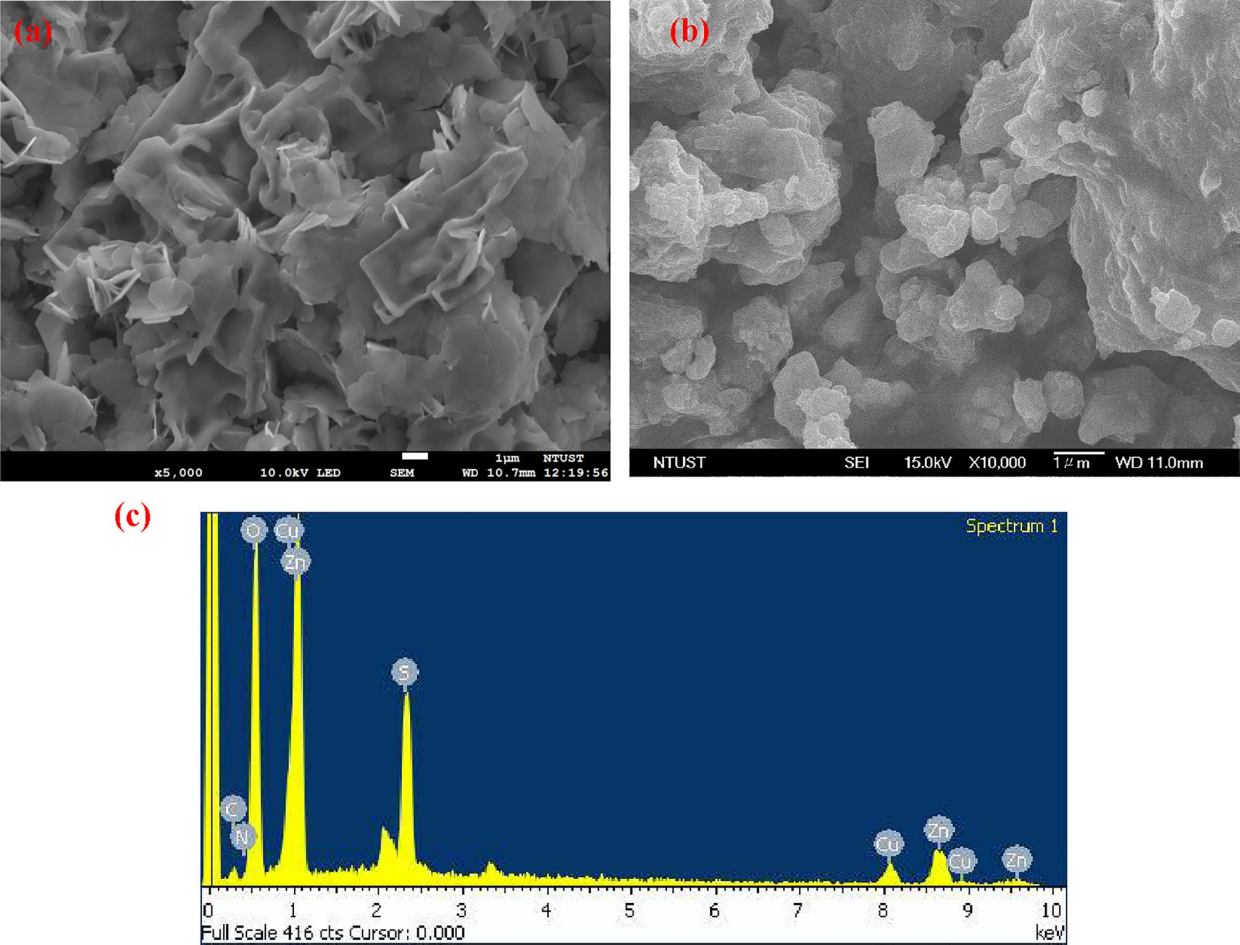
SEM image of (**a**) ZnO NPs and (**b**) ZnO/20% Cu-DPA nanocomposite (**c**) EDS spectra of ZnO/20% Cu-DPA nanocomposite.

The surface morphologies of the samples were also verified via SEM analysis. In Fig. 2, we have a SEM image of a sample that consists of ZnO NPs and a ZnO/20% Cu-DPA composite. From Fig. 2a, we can observe the surface morphologies of aggregated ZnO plate NPs arising due to polarity and electrostatic attraction of ZnO NPs^40^. Adding surfactants or other organic molecules to the reaction cell is one strategy for preventing this aggregation^34^. The SEM images of the ZnO/Cu-DPA sample (Fig. 2b) reveal that the agglomeration was decreased due to the presence Cu-DPA complex. Moreover, Fig. 2c shows the EDS spectra clearly showed Cu, Zn, N, C, and O elements from ZnO/Cu-DPA sample. The element S was detected as impurity.

**Figure 3:**
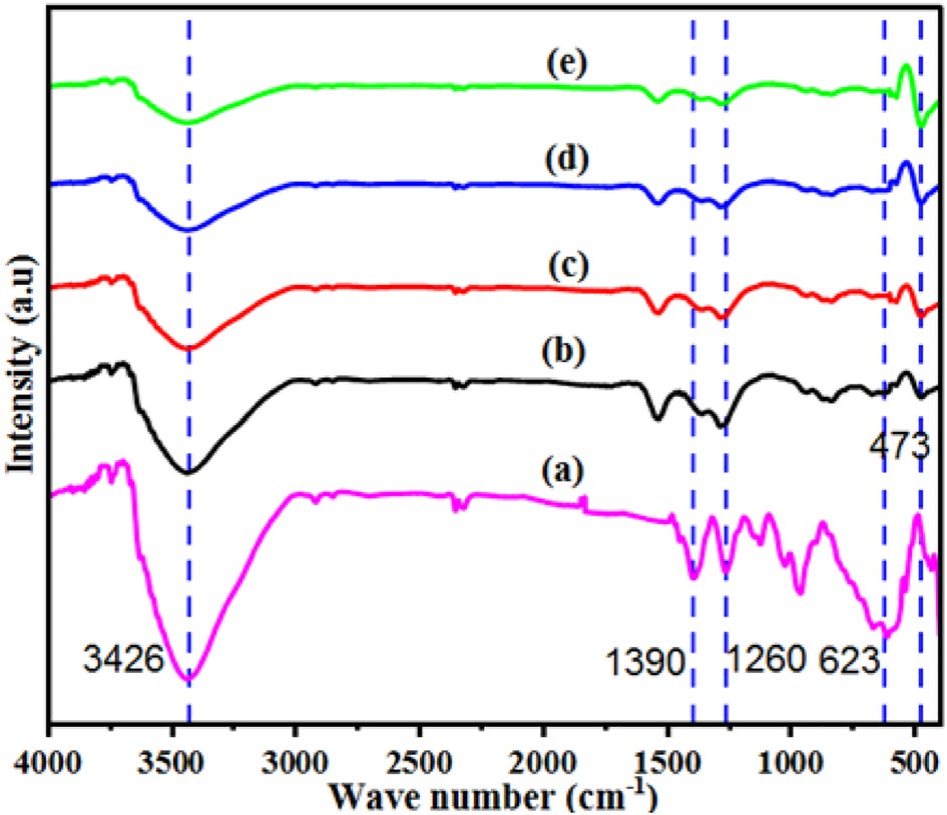
FT-IR spectra of (**a**) Cu-DPA, (**b**) ZnO/20% Cu-DPA, (**c**) ZnO/30% Cu-DPA, (**d**) ZnO/40% Cu-DPA and (**e**) ZnO/50% Cu-DPA.

To investigate the structural information of the synthesized material, we analyzed FTIR spectra of the produced Cu-DPA, ZnO, and ZnO/20% Cu-DPA composites; the results are shown in Fig. 3. The as-prepared Cu-DPA sample complex has a significant peak at 3426 cm^−1^ (Fig. 3a), which may be related to N–H stretching (a). The N–H stretching vibrations of the aromatic ring are ascribed to the 1390 and 1260 cm^−1^ peaks. At 954 cm^−1^, DPA ligand asymmetric stretching with Cu is found^26^. This diminished characteristic of the peak is a solid indicating the bond created between Cu and the DPA ligand. At 623 cm^−1^, a Cu_2_O peak was also seen. It agrees well with the XRD findings and corresponds to previously reported studies^41^. Figure 3b–e shows the FTIR spectra of ZnO/Cu-DPA composites at different ratios, where an extra peak is seen at 473 cm^−1^ because of Zn–O stretching. According to the results of another investigation, ZnO has FTIR absorption peaks at 551 and 889 cm^−1^^42^. It has also been reported that the FTIR absorption of ZnO NPs has a peak at around 451 cm^−1^^43^. Additionally, ZnO species found to have an absorption peak about 454 cm^−1^ and a shoulder near 545 cm^−1^^44^. Therefore, these studies improve the existence of ZnO species.

### Optical properties of ZnO/Cu-DPA composite catalysts

**Figure 4:**
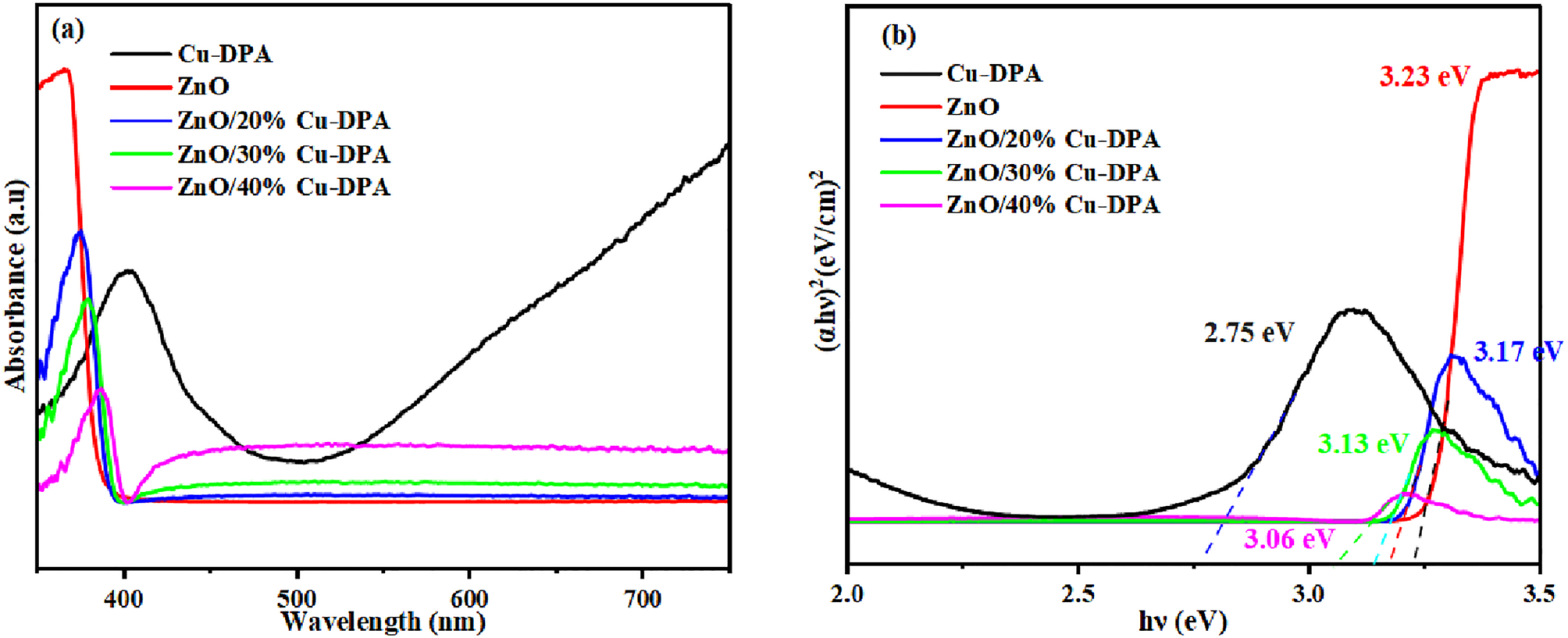
(**a**) Ultraviolet–visible absorption spectrum and (**b**) the (αhv)^2^-hv plot from the ultraviolet–visible absorption spectrum.

The absorption spectra of Cu-DPA, ZnO, and the resulting ZnO/Cu-DPA composite were recorded using absorbance spectroscopy, and the results are shown in Fig. 4. The as synthesized Cu-DPA, ZnO, ZnO/20%Cu-DPA, ZnO/30%Cu-DPA, and ZnO/40%Cu-DPA samples had the λ_max_ values of 412, 366, 380, 388, and 402 nm, respectively (Fig. 4a). According to the findings, coupling ZnO with Cu-DPA altered the absorption in the direction of longer wavelengths. In contrast, the composite significantly improved visible light harvesting compared to ZnO alone. The optical energy bandgap of all synthesized samples was calculated using Tauc plots (Fig. 4b) by extrapolating of absorption edge by using the following Eq. (3) considering direct allowed transitions^45^.

3 $$\left( {\alpha {\text{hv}}} \right)^{2} = {\text{k}}\left( {{\text{hv}} - {\text{E}}_{{\text{g}}} } \right)$$

where α is the absorption coefficient, h is Planck’s constant, k is the absorption constant for a direct transition, and ν is wave number. The bandgap of ZnO, Cu-DPA, ZnO/20%Cu-DPA, ZnO/30%Cu-DPA and ZnO/40%Cu-DPA is 3.23, 2.75, 3.17, 3.13, and 3.06 eV, respectively. This bandgap data reveals a significant decrease in the bandgap of composites upon the incorporation of ZnO NPs in Cu-DPA. This decrease in bandgap improves the absorption of visible light, reduces the electron–hole pair recombination, and improves the photocatalytic activity photodegradation of MB.

### Photocatalytic activities of composite photocatalysts

Recently, visible light-mediated photocatalysts (e.g., copper(I) oxide^46^, zinc oxide and ZnO/Cu_2_O nano-catalyst 10) have been demonstrated to be powerful candidate for photo-catalytic degradation of methylene blue (MB) in an aqueous solution^46,47^. As a result, ZnO/Cu-DPA nanocomposite would provide a relatively high photocatalytic activity compared to an individual ZnO and Cu-DPA. The catalytic efficiency of ZnO/Cu-DPA nanocomposite was further assessed by catalytic reduction of MB. Thus, we incubated ZnO/Cu-DPA nanocomposite with MB under exposure to visible light.

**Figure 5:**
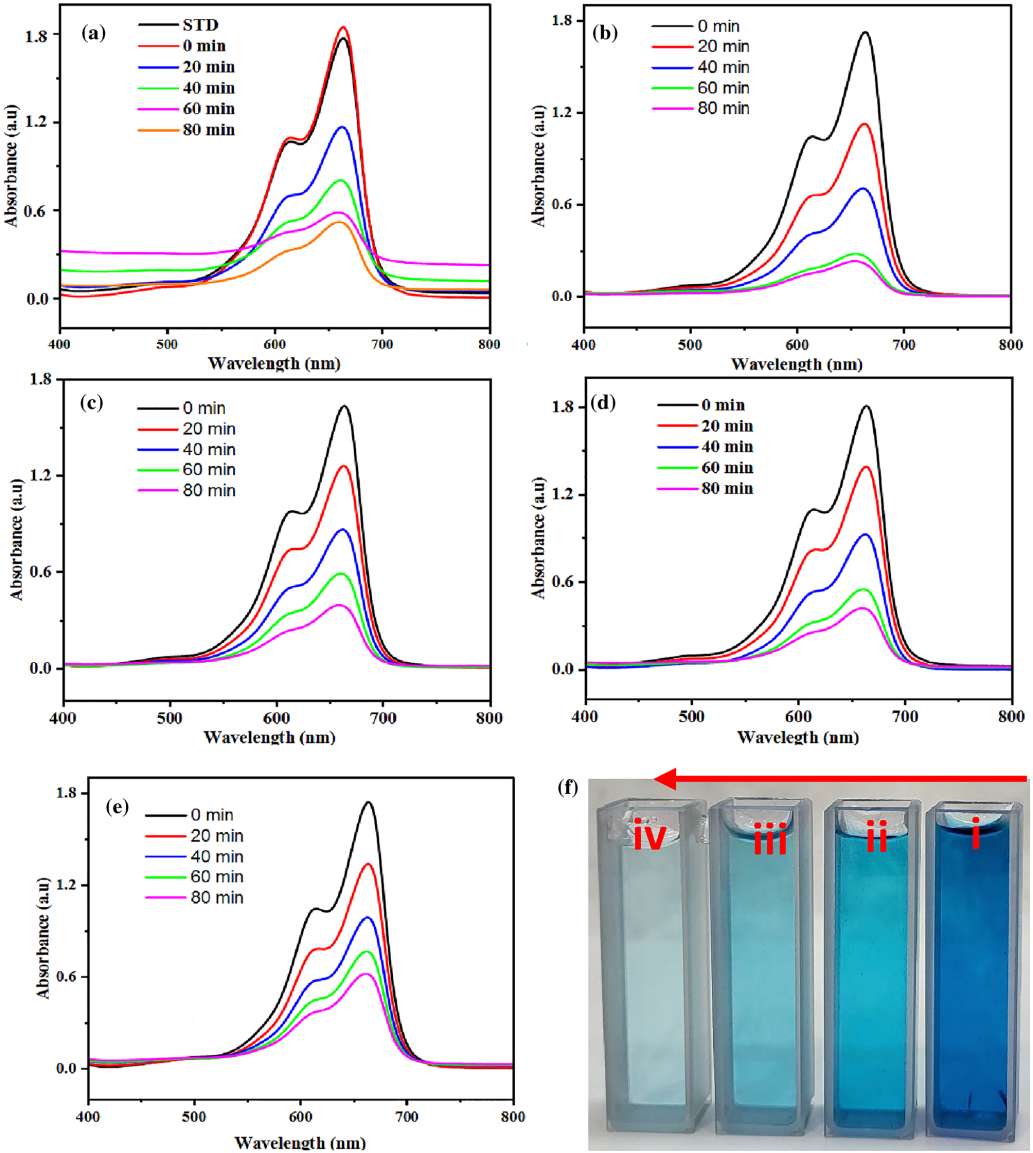
UV–Visible absorption spectra of MB after different irradiation times using (**a**) ZnO (**b**) ZnO/20% Cu-DPA, (**c**) ZnO/30% Cu-DPA, (**d**) ZnO/40% Cu-DPA and (**e**) ZnO/50% Cu-DPA catalysts (**f**) photographs of MB before [i] and after incubation [ii, iii, iv] of ZnO/20% Cu-DPA.

As shown in Fig. 5, the synthesized samples effectively degraded MB, and the concentration of MB decreased over time. The degrading process was placed in the open air with visible light for 80 min with a 20-min gap between each exposure. After 80 min, all of the synthesized photocatalysts successfully degraded the MB. However, ZnO/20%Cu-DPA exhibits the highest photocatalytic activity towards methylene blue degradation compared to all other synthesized samples. Figure 5b also shows that after 80 min, the dye solution containing ZnO/20%Cu-DPA has the lowest absorbance, in contrast to the dye solution with all other synthesized samples.

The % degradation (D) of MB is estimated by using the below equation.

4 $${{\% D}} = \frac{{{\text{Ao}} - {\text{At }}}}{{{\text{Ao}}}}{ } \times 100{{\% }}$$

Here At is absorbance at t min and A_0_ is absorbance at t = 0 min.

The calculated % of degradation is 71.80, 87.39, 78.57, 77.11 and 66.17% for ZnO, ZnO/20%Cu-DPA, ZnO/30%Cu-DPA, ZnO/40%Cu-DPA and ZnO/50%Cu-DPA, respectively (Fig. 5a–e). Except for ZnO/50%Cu-DPA (Fig. 5e), all composites have higher degradation efficiency than ZnO alone. Among the composites, ZnO/20%Cu-DPA has a higher efficiency (Fig. 5b). The low effectiveness of the ZnO NPs is a result of their larger bandgap energy. Thus, small amounts of electrons from ZnO NPs can be excited by the irradiated photons, and thus a low amount of e−/h+ pairs and other reactive species can be produced. The enhanced composite effect may be linked to the enhanced e−/h+ pair separation. The Cu-DPA coupling improved ZnO optical characteristics, and a red shift in the bandgap of coupled system was detected. The effect of varying the ZnO/Cu-DPA ratio on the photocatalytic activity of the resulting composites was investigated, and the results are depicted in Fig. 5. The findings demonstrate that the photocatalytic activity of the composite is sensitive to variations in its compositional ratio. This further verifies the correlation between the composite component ratio and the rate of e−/h+ recombination. With the ratio ranging from 80 to 20% Cu-DPA, the best matching between the components was achieved to achieve the maximum inhibition of e/h recombination. The photographs in Fig. 5f show before incubation of the nanocatalyst and after incubation of ZnO/20%Cu-DPA in MB solution, which is consistent with the absorbance spectra results. According to Table S1, the % degradation of ZnO/20%Cu-DPA is superior and equivalent to that of previously reported photocatalysts.

### Kinetic study of MB dye degradation

**Figure 6:**
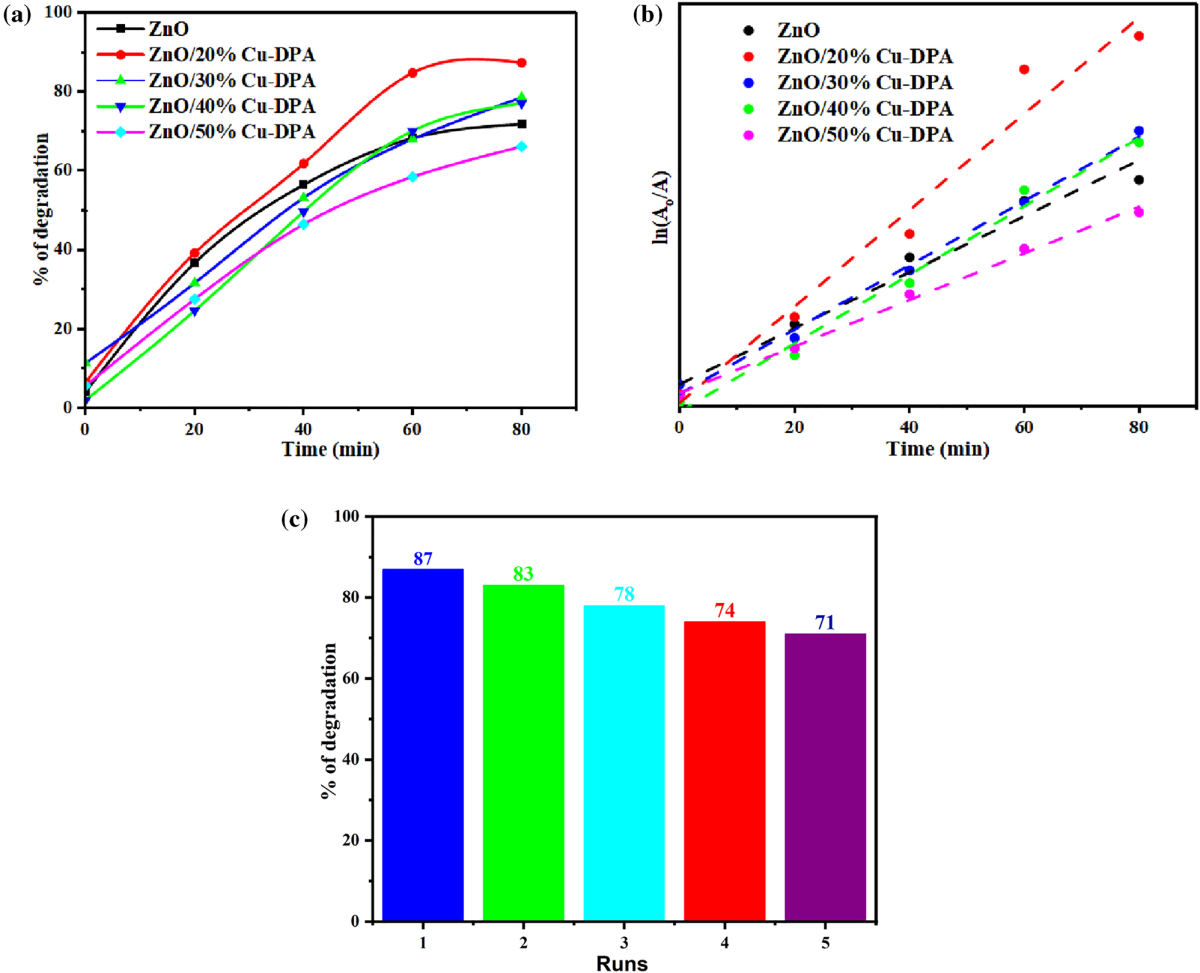
(**a**) % of degradation of MB using the synthesized catalyst in different irradiation times, (**b**) The first-order kinetic plot at different irradiation times, (**c**) The reusability efficiency for ZnO/20% Cu-DPA catalyst in three cycles.

The photodegradation rate of MB in a typical heterogeneous photocatalytic process could be predicted by pseudo-first-order kinetics. A pseudo-first-order reaction is a second-order reaction that exhibits the characteristics of a first-order reaction. This reaction occurs when one of the reacting materials is substantially more abundant than the other. There is little sensitivity to changes in their concentrations in the reaction. Therefore, the reaction is now solely dependent on the concentration of the individual reactant^48^. In the rate law, the concentration of the other reactant is assumed to be constant. As a result, the reaction order becomes one. Because of this, variation in the concentration of MB during photodegradation affected the reaction rate, whereas the amount of water (source of free radicals) did not. Photodegradation experiments were performed on MB solutions, and Spectra of ultraviolet–visible light were recorded as a function of exposure time (Fig. 4). (A/Ao) values were recorded as a measure of degraded MB molecules from the absorption maxima of the recorded spectra, and then a plot of ln (A/Ao) versus time was constructed to study the kinetics of the MB photodegradation process (Fig. 6). The kinetic rate removal of MB dye was demonstrated using Eq. (5)

5 $${\text{ln}}\frac{{{\text{At}}}}{{{\text{A}}_{0} }} = - {\text{kt}}$$

where A_0_ and A_t_ are the initial concentration and the concentration at a time (t), respectively, and k is the rate constant. As displayed in Fig. 6a, change in the ratio of the composite changes its photocatalytic activity which agreed with the absorbance behaviours of the catalysts. As shown in Fig. 6b, the MB degradation indicates the pseudo-first-order kinetics^49^. The constants of estimated rates of ZnO, ZnO/20%Cu-DPA, ZnO/30%Cu-DPA, ZnO/40%Cu-DPA and ZnO/50%Cu-DPA were 0.016, 0.023, 0.019, 0.018 and 0.014 min^−1^, respectively. According to the findings, the best rate constant was found at ZnO/20%Cu-DPA catalysts. The reusability of the most efficient sample, ZnO/20% Cu-DPA photocatalyst, was investigated by continuously monitoring the photo-degradation of MB under visible light irradiation after three cycles. Figure 6c shows that photodegradation of a ZnO/20%Cu-DPA nanocomposite results in 87% MB degradation in the first cycle. In the second, third, fourth and fifth cycles, the calculated photocatalytic activity of the sample was 83%, 78%, 74 and 71%, respectively. This means that after five cycles, there is only a 16% decrease in photocatalytic efficiency. It is believed that the inactivation of some adsorption sites on the photocatalyst's surface is responsible for the modest reduction in photocatalytic activity and/or the dye solution undergoing a partial phase change catalyzed by a photocatalyst in the presence of visible light. Because no significant decrease in photocatalytic activity is observed during successive cycles, the ZnO/20% Cu-DPA sample can be photo-catalytically reusable. In addition, the rate constant for the photodegradation of MB using a ZnO/Cu-DPA nanocomposite is higher than that reported for other nanomaterials (Table S2). The results show that a ZnO/Cu-DPA nanocomposite efficiently triggers the photodegradation of MB into low molecular products.

To determine the role of specific active species in the photocatalytic progression, 0.1 M isopropanol, EDTA, and Cu(NO_3_)_2_ were added to the MB solution as chemical selective scavengers for hydroxyl radicals, holes, and electrons, respectively. Figure S3 depicts the result, which shows that the photocatalytic activity of ZnO/20% Cu-DPA is reduced in the control experiment. The results show that isopropanol, Cu(NO_3_)_2_, EDTA scavengers, and no scavengers cause 21%, 25%, 59%, and 87% degradation, respectively. The results show that the least degradation occurred when isopropanol, an OH· scavenger, was present. This indicated that OH· is the most important specific active radical in the photocatalytic degradation of MB.

Figure S4 demonstrates that the synthesized ZnO/Cu-DPA nanocomposite efficiently transforms MB into dissolved O_2_ into OH· suggesting it may be a viable alternative visible light-mediated photocatalyst for MB degradation. During the photocatalytic experiments, the reactive oxygen species of ·OH were generated, which are highly responsive to photocatalytic degradation of MB. Further verification of the oxidative radicals in the ZnO/Cu-DPA nanocomposite system for the degradation of MB was achieved through an EPR analysis using a spin-trapping agent, specifically 3,4-dihydro-2,3-dimethyl-2H-pyrrole 1-oxide (DMPO). There was no EPR signal was observed in the absence of visible light after 80 min of mixing ZnO/Cu-DPA nanocomposite with DMPO in the presence of dissolved O_2_ (Figure S4). When the ZnO/Cu-DPA nanocomposite was exposed to visible light under the same reaction conditions, the EPR spectra displayed a four-line hyperfine splitting with intensity ratios of 1:2:2:1 (Figure S4), suggesting the development of DMPO-OH adducts. In photocatalytic systems, the presence of ·OH is indicated by the production of DMPO-OH adducts^50,51^.

**Figure 7:**
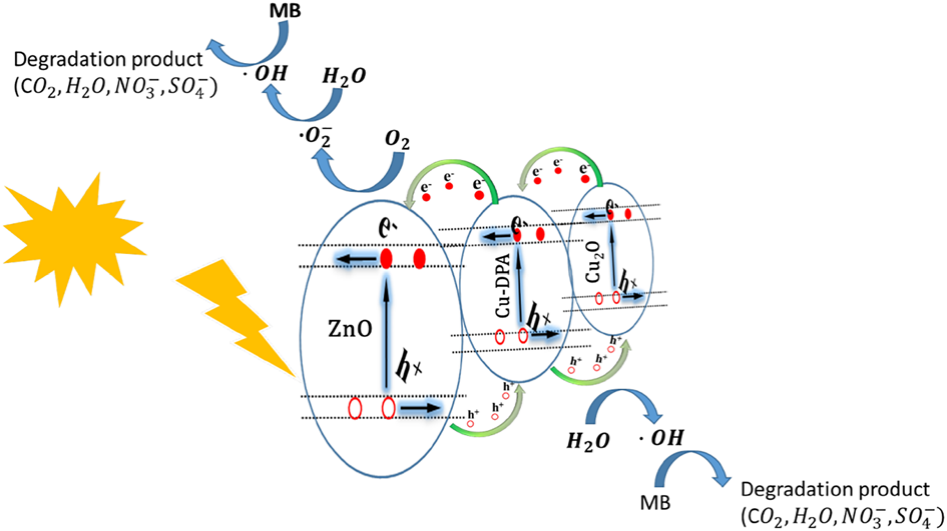
Proposed mechanism for photocatalytic-degradation of MB using ZnO/20% Cu-DPA.

A schematic depiction of the photodegradation mechanism of MB by the ZnO/Cu-DPA nanocomposite is presented in Fig. 7. As revealed on XRD diffraction (Figure S1) and FTIR spectra, there is a formation of Cu_2_O during the synthesis of Cu-DPA. The same result was also displayed in other literature^32^. As a p-type semiconductor, Cu_2_O exhibits modest visible light activity^52^. However, in ZnO/Cu-DPA nanocomposite, because of its high absorption of visible light, Cu_2_O plays a crucial role in photocatalytic activity by facilitating maximum electron–hole separation. In addition, heterostructure with ZnO prevents the photogenerated charge carrier from recombining. The photocatalytic activity of the nanocomposite was enhanced because the photo-excited charges were confined at intrinsic defect sites in the ZnO structure^53^. More specifically, ZnO has been found to have three key defects: hole acceptors (h+), electron acceptors (e−), and oxygen vacancy. Consequently, these defects can serve as sub-bandgap states with energies lower than ZnO’s true bandgap, which might be another source for absorbing visible light^54^. This boosts the possibility for photocatalytic reduction and oxidation reactions. According to the absolute electronegativity of ZnO and Cu_2_O, the edge positions of the valence band (VB) and conduction band (CB) were calculated. According to previous research, the electronegativity and the energies of the conduction (E_CB_) and valence (E_VB_) band edges can be represented as follows^55^.

6 $${\text{E}}_{VB} = \chi - {\text{E}}_{e} + 0.{\text{5E}}_{g}$$

7 $${\text{E}}_{CB} = {\text{E}}_{VB} {-}{\text{E}}_{g}$$

where χ is the absolute electronegativity of the semiconductor expressed as the geometric mean of the absolute electronegativity of the constituent atoms, E_e_ is the energy of free electrons in the hydrogen scale (∼ 4.5 eV) and E_g_ is the energy bandgap of the semiconductor. The χ values for ZnO and Cu_2_O are 5.79 and 5.32 eV^56,57^. The bandgap of ZnO and Cu_2_O are 3.23 and 1.88 eV. Table S3, summarizes the calculated value of E_*VB*_ and E_*CB*_ of ZnO, Cu-DPA, and Cu_2_O calculated using Eqs. (6, 7).

## Conclusions

In summary, we have successfully synthesized ZnO/Cu-DPA nanocomposite to decompose MB dye. As far as we know, this is the first effort to couple Cu-DPA complex with ZnO NPs using a simple approach to apply as an effective photocatalyst under visible light. Using different ratios of Cu-DPA coupling with ZnO NPs, the bandgap of ZnO was modified and exhibited enhanced photodegradation of MB. ZnO/20%Cu-DPA coupled system displayed the best efficiency (87%) for photodegradation of MB within 80 min when exposed to visible light. However, ZnO, ZnO/30%Cu-DPA, ZnO/40%Cu-DPA, and ZnO/50%Cu-DPA degradation efficiencies were 71.80, 78.57, 77.11, and 66.17%, respectively. ZnO/20% Cu-DPA reusability, as the most efficient photocatalyst, was investigated across three cycles. Since the synthesized ZnO/Cu-DPA nanocomposite efficiently converts dissolved O2 into ·OH, it can potentially be a replacement visible light-mediated photocatalyst for MB degradation.

### Supplementary Information

Supplementary Information.

## Data Availability

The datasets used and/or analyzed in this study are accessible in the publication and can be obtained upon request from the corresponding author.
