# Supplementary material for: Highly efficient visible light active ZnO/Cu-DPA composite photocatalysts for the treatment of wastewater contaminated with organic dye
Source: Sci Rep. 2023 Sep 30;13:16454. doi: 10.1038/s41598-023-43842-z (PMC10542347; doi:10.1038/s41598-023-43842-z)
Supplement: Supplementary file 1 — Supplementary Information. [file 41598_2023_43842_MOESM1_ESM.docx]

**Highly Efficient Visible Light Active ZnO/Cu-DPA composite Photocatalysts for the Treatment of Wastewater Contaminated with Organic Dye**

Biniyam Abdu Berehe,^a^ Ayalew H.Assen,^a^ A. Santhana Krishna Kumar,^b,c^ Hidayath Ulla,^d^ Alemayehu Dubale Duma,^e^ Jia-Yaw Chang,^f^ Gangaraju Gedda ^g,h^* and Wubshet Mekonnen Girma ^a^*

^a^ Department of Chemistry, College of Natural Science, Wollo University, P.O. Box:1145, Dessie, Ethiopia

^b^ Department of Chemistry, National Sun Yat-sen University, No. 70, Lien-hai Road, Gushan District, Kaohsiung 80424, Taiwan.

^c^ Faculty of Geology, Geophysics and Environmental Protection, AGH University of Science and Technology, Al. Mickiewicza 30, 30-059, Krakow, Poland.

^d^ Department of Physics, School of Engineering, Presidency University, Bangalore 560064, India

^e^ Nanotechnology Directorate, Bio and Emerging Technology Institute (BETin), P.O.Box 5954, Addis Ababa, Ethiopia

^f^ Department of Chemical Engineering, National Taiwan University of Science and Technology, Taipei, Taiwan, Republic of China

^g^ Central Research Laboratory, K S Hegde Medical Academy, NITTE (Deemed to be University), Deralakatte, Mangaluru -575018, Karnataka, India.

^h^ Department of Animal Science & Technology and BET Research Institute, Chung-Ang University, Anseong, Gyeonggi-do 17546, Republic of Korea.

*Corresponding authors: Gangaraju Gedda and Wubshet Mekonnen Girma

Department of Chemistry, College of Natural Science, Wollo University, P.O. Box:1145, Dessie, Ethiopia

E-mail: [wubshet.mekonnen@wu.edu.et](mailto:wubshet.mekonnen@wu.edu.et)

Tel.: +251-910804026.

**Supporting information**


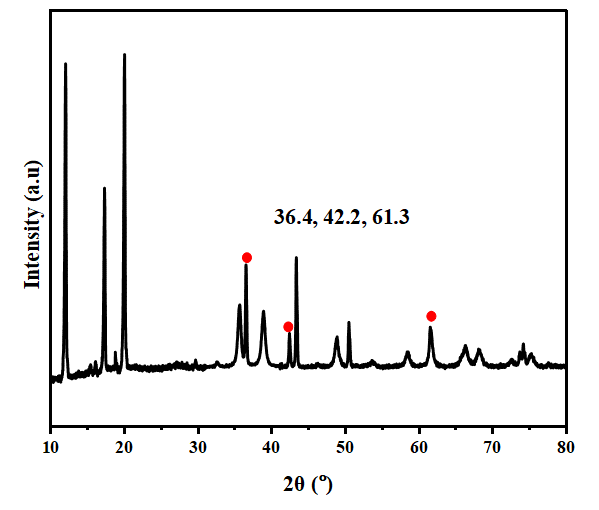


**Figure S1: XRD patterns of Cu-DPA**

**Figure S2:** The W–H plot used for the estimation of the crystallite sample (a) Cu-MOF, (b) ZnO, (c) ZnO/20% Cu-DPA, (d) ZnO/30% Cu-DPA, (e) ZnO/40% Cu-DPA and (f) ZnO/50% Cu-DPA


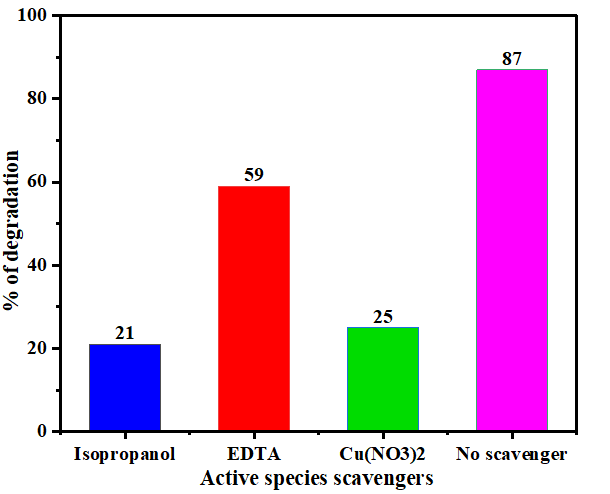


**Figure S3:** Degradation percentage of MB in the presence of different scavengers


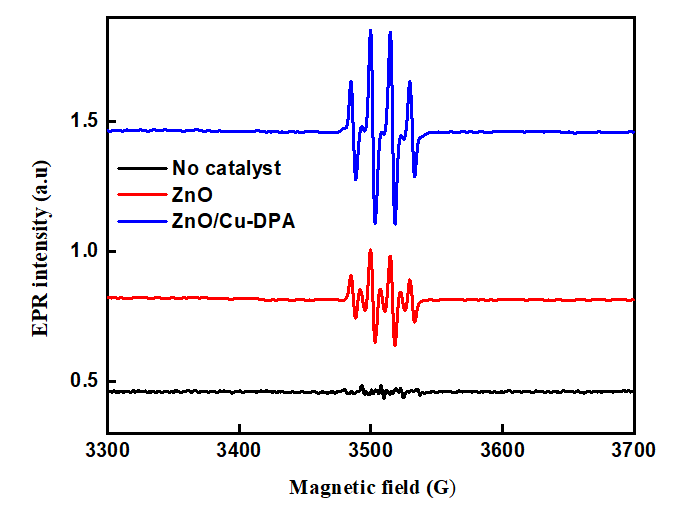


**Figure S4:** The EPR spectra of a solution (pH 6.5) containing 200 mg mL ^-1^ of photocatalyst ZnO/20% Cu-DPA nano-composite, 100 mM DMPO, 100 mM phosphate buffer and 50% of DMSO before (blak line) and after (blue line) the irradiation of visible light.

| Material/catalyst | % of degradation | References |
| --- | --- | --- |
| Cr_2_O_3_/ZnO | 85% | [^1^](#_ENREF_1) |
| TiO_2_/Al_2_O_3_@Cu(BDC) | 37.77% | [^2^](#_ENREF_2) |
| Cu_2_O–ZnO | 78.0% | [^3^](#_ENREF_3) |
| MgFe_2_O_4_/ZnO | 74% | [^4^](#_ENREF_4) |
| WO_3_.NiO.ZnO | 66.19% | [^5^](#_ENREF_5) |
| ZnO/Cs_4_SiW_12_O_40_ | 76.9% | [^6^](#_ENREF_6) |
| ZnO/20%Cu-DPA | 87% | **This work** |

**Table S1:** Comparison of % degradation of ZnO/20%Cu-DPA with previously reported catalysts

**Table S2.** Comparison of catalytic activities of ZnO/20%Cu-DPA with catalysts for reduction of MB reported in literatures

| **S/N** | **Catalytic name** | **Time K(min^-1^)** | **Rate constant K** | **Ref** |
| --- | --- | --- | --- | --- |
| 1 | Nitrogen-doped zinc oxide (N-ZnO), | 60 | 0.0202 min^-1^ | [^7^](#_ENREF_7) |
| 2 | Nitrogen-doped zinc oxide@carbon dots (CDs) | 60 | 0.0299 min^-1^ | [^7^](#_ENREF_7) |
| 3 | Fe–ZnO nanomaterials | 180 | 0.0106 min^-1^ | [^8^](#_ENREF_8) |
| 4 | CuO nano-composite | 110 | 0.0025 min^-1^ | [^9^](#_ENREF_9) |
| 5 | ZnO nano-composite | 110 | 0.0036 min^-1^ | [^9^](#_ENREF_9) |
| 6 | ZnO/CuO nano-composite | 110 | 0.0148 min^-1^ | [^9^](#_ENREF_9) |
| 7 | ZnO thin film | 540 | 1.54 X10^-9^ | [^10^](#_ENREF_10) |
| 8 | Zinc oxide nanoparticles | 90 | 5.20 X10^-4^ Sec^-1^ | [^11^](#_ENREF_11) |
| 9 | ZnO–reduced graphene oxide–carbon nanotube composites | - | 0.011 min^-1^ | [^12^](#_ENREF_12) |
| 10 | Graphene-zinc oxide nanorod nanocomposite | 90 | 10.7 X 10^4^  Sec^-1^ | [^13^](#_ENREF_13) |
| 11 | ZnO–reduced graphene oxide | 130 | 0.0117 min^-1^ | [^14^](#_ENREF_14) |
| 12 | Mesoporous zinc oxide (ZnO) @ reduced graphene oxide (ZnO/rGO hybrids) | 220 | 2.831 h^−1^ | [^15^](#_ENREF_15) |
| 13 | Graphdiyne-ZnO Nanohybrids | 120 | 0.00426 min^-1^ | [^16^](#_ENREF_16) |
| 14 | ZnO | 80 | 0.0016 min^-1^ | This study |
| 15 | ZnO/20%Cu-DPA | 80 | 0.023 min^-1^ | This study |

Table S3. Absolute electronegativity, E*_g_*, *E_VB_,* and E_CB_ values for ZnO and CuO (vs NHE).

| Semiconductor | ꭓ (eV) | E_g_ (eV) | E_VB_ (eV) | E_CB_(eV) |
| --- | --- | --- | --- | --- |
| ZnO | 5.79 | 3.23 | 0.945 | -2.285 |
| Cu_2_O | 5.32 | 1.88 | 1.76 | 0.395 |
| Cu-DPA | 2.078 | 3.17 | -2.677 | -5.847 |

**References**

1 Zelekew, O. A., Fufa, P. A., Sabir, F. K. & Duma, A. D. Water hyacinth plant extract mediated green synthesis of Cr2O3/ZnO composite photocatalyst for the degradation of organic dye. *Heliyon* **7**, e07652 (2021).

2 Jatoi, Y. F., Fiaz, M. & Athar, M. Synthesis of efficient TiO2/Al2O3@Cu(BDC) composite for water splitting and photodegradation of methylene blue. *Journal of the Australian Ceramic Society* **57**, 489-496, doi:10.1007/s41779-020-00548-z (2021).

3 Norouzi, A., Nezamzadeh-Ejhieh, A. & Fazaeli, R. A Copper (I) oxide-zinc oxide nano-catalyst hybrid: Brief characterization and study of the kinetic of its photodegradation and photomineralization activities toward methylene blue. *Materials Science in Semiconductor Processing* **122**, 105495 (2021).

4 Arshad, J. *et al.* Integration of 2D graphene oxide sheets with MgFe2O4/ZnO heterojunction for improved photocatalytic degradation of organic dyes and benzoic acid. *Ceramics International* **49**, 18988-19002 (2023).

5 Abo-Dief, H. M. *et al.* Ternary metal oxide WO3. NiO. ZnO nanoparticles and their composite with CNTs for organic dye photocatalytic degradation. *Ceramics International* **48**, 22269-22277 (2022).

6 Bai, L. *et al.* Sunlight-driven photocatalytic degradation of organic dyes in wastewater by chemically fabricated ZnO/Cs4SiW12O40 nanoheterojunction. *Applied Surface Science* **599**, 153912 (2022).

7 Ayu, D. G. *et al.* Photocatalytic Degradation of Methylene Blue Using N-Doped ZnO/Carbon Dot (N-ZnO/CD) Nanocomposites Derived from Organic Soybean. *ACS omega* **8**, 14965-14984 (2023).

8 Masombaigi, H., Rezaee, A. & Nasiri, A. Photocatalytic degradation of Methylene Blue using ZnO nano-particles. *Iranian Journal of Health and Environment* **2**, 188-195 (2009).

9 Basit, R. A. *et al.* Successive photocatalytic degradation of methylene blue by ZnO, CuO and ZnO/CuO synthesized from coriandrum sativum plant extract via green synthesis technique. *Crystals* **13**, 281 (2023).

10 Kulis-Kapuscinska, A. *et al.* Photocatalytic degradation of methylene blue at nanostructured ZnO thin films. *Nanotechnology* (2022).

11 Venkatesan, S. *et al.* Methylene blue dye degradation potential of zinc oxide nanoparticles bioreduced using Solanum trilobatum leaf extract. *Results in Chemistry* **4**, 100637 (2022).

12 Lv, T., Pan, L., Liu, X. & Sun, Z. Enhanced photocatalytic degradation of methylene blue by ZnO–reduced graphene oxide–carbon nanotube composites synthesized via microwave-assisted reaction. *Catalysis Science & Technology* **2**, 2297-2301 (2012).

13 Nipane, S., Korake, P. & Gokavi, G. Graphene-zinc oxide nanorod nanocomposite as photocatalyst for enhanced degradation of dyes under UV light irradiation. *Ceramics International* **41**, 4549-4557 (2015).

14 Zhou, X., Shi, T. & Zhou, H. Hydrothermal preparation of ZnO-reduced graphene oxide hybrid with high performance in photocatalytic degradation. *Applied surface science* **258**, 6204-6211 (2012).

15 Shanmugasundaram, A. *et al.* Facile in-situ formation of rGO/ZnO nanocomposite: Photocatalytic remediation of organic pollutants under solar illumination. *Materials Chemistry and Physics* **218**, 218-228 (2018).

16 Thangavel, S. *et al.* Graphdiyne–ZnO nanohybrids as an advanced photocatalytic material. *The Journal of Physical Chemistry C* **119**, 22057-22065 (2015).
